# Surgical treatment of thoracic aortic pseudoaneurysm caused by *Brucella melitensis*

**DOI:** 10.1186/s13019-023-02171-y

**Published:** 2023-02-14

**Authors:** Hong-Wei Guo, Yang-Xue Sun, Jing Sun, Xiang-Yang Qian

**Affiliations:** Department of Surgery, Fuwai Hospital, National Center for Cardiovascular Diseases, Chinese Academy of Medical Sciences and Peking Union Medical College, 167# Beilishi Road, Beijing, 100037 China

**Keywords:** Thoracic aorta, Pseudoaneurysm, *Brucella melitensis*, Surgical procedure, Relapse

## Abstract

Thoracic aortic pseudoaneurysm caused by *Brucella melitensis* is extremely rare with extremely few cases reported to date. Herein, we present the case of a 65 year-old man with a huge pseudoaneurysm of the proximal descending thoracic aorta, involving the left subclavian artery and distal arch. Surgery was performed to replace the proximal descending aorta with a self-made bovine pericardial duct and the left subclavian artery with a 10 mm artificial vessel under deep hypothermic circulatory arrest; the patient recovered uneventfully. However, continued follow-up is required for long-term results.

## Introduction

Brucellosis is a zoonotic disease, resulting in thoracic aortic pseudoaneurysm, an extremely rare condition. We reported a case of thoracic aortic pseudoaneurysm caused by recurrent brucellosis post-treatment. Surgery was performed to replace the distal arch and proximal descending aorta with a self-made bovine pericardial duct and the left subclavian artery with a 10 mm artificial vessel. The patient achieved good short-term results.

## Case report

**Fig. 1:**
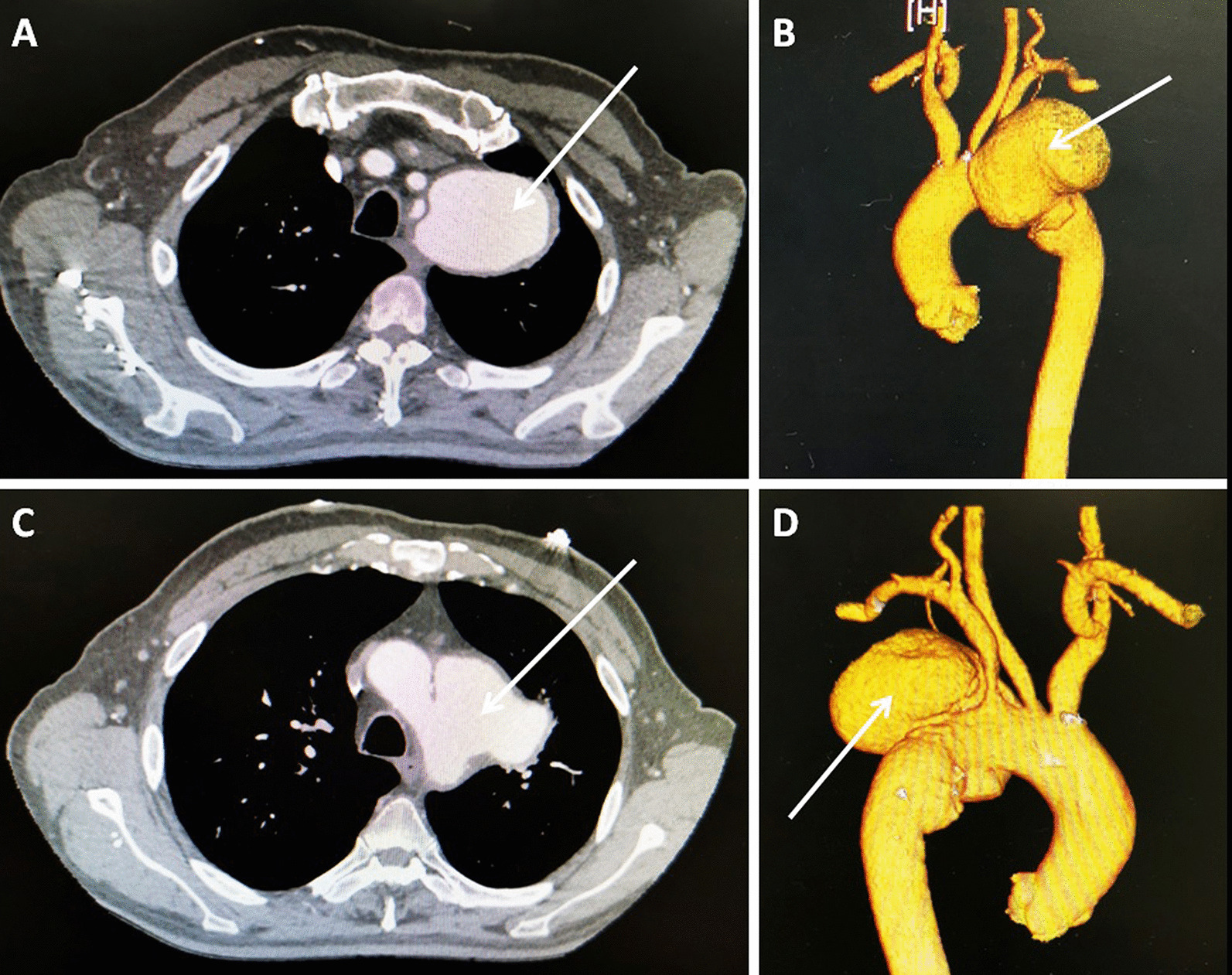
Preoperative computed tomographic scans showing a huge aortic pseudoaneurysm (58 × 56 mm, arrow indicating) involving the distal arch, proximal descending aorta, and left subclavian artery. **A** and **C **The computed tomography of aortic pseudoaneurysm. **B** Front view. **D** Posterior view

A 65 year-old man was admitted to our hospital with a 2 month history of intermittent fever and hoarseness. The patient suffered from brucellosis 8 years ago and recovered post-treatment. Then, he previously engaged in the slaughtering industry. *Brucella* agglutination test was positive. A computed tomographic (CT) scan showed a huge pseudoaneurysm of the proximal descending thoracic aorta, involving the left subclavian artery and distal arch (Fig. 1). Hematologic investigations show CRP, ESR and RF increased. And the *Brucella* agglutination test shows positive. So, the treatment with doxycycline, ceftriaxone and amikacin for one month started it here, and the next treatment with doxycycline and rifampicin for two months preoperative.

**Fig. 2:**
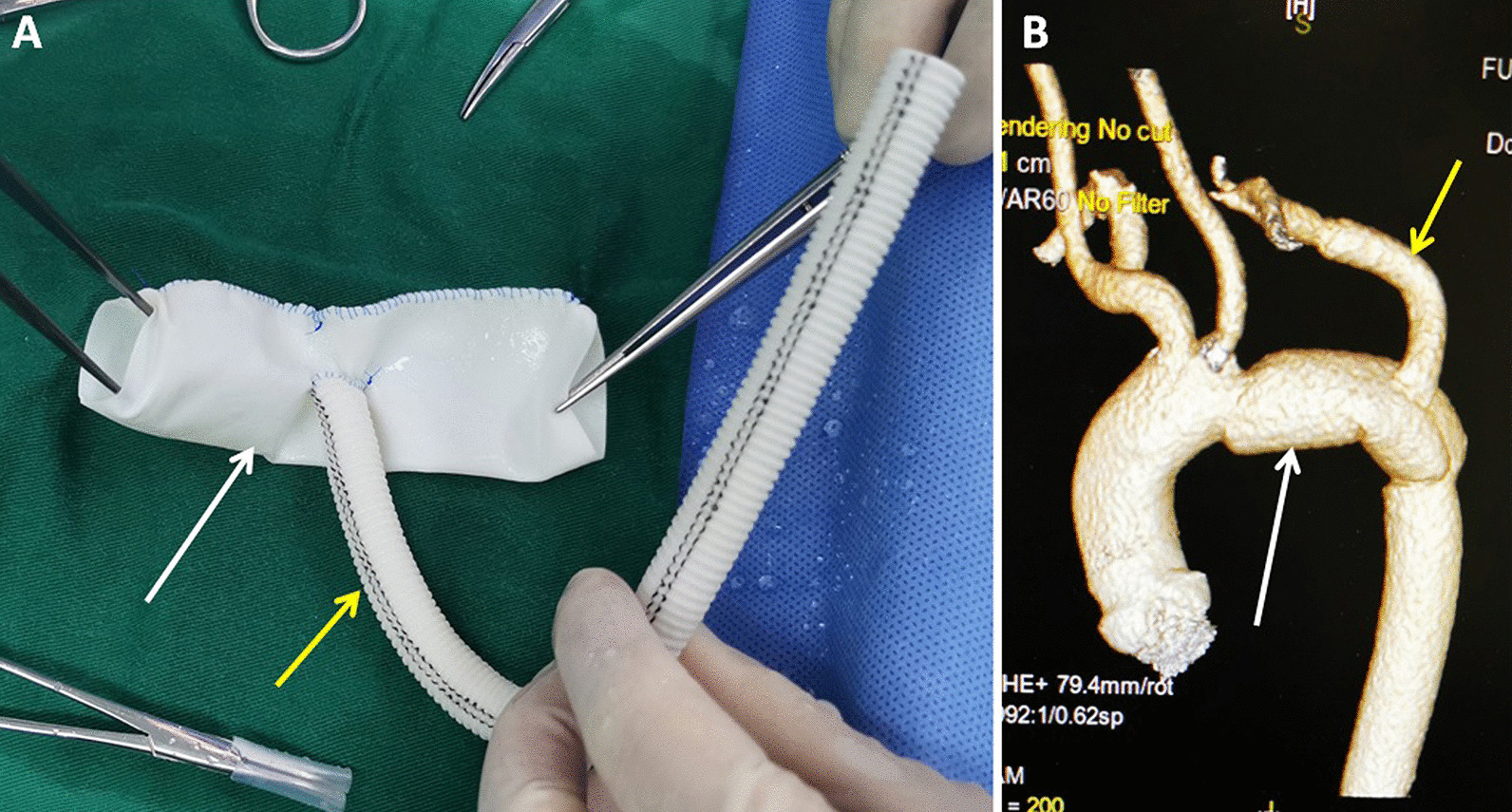
**A** A 24 mm-diameter and 120 mm-long duct (white arrow indicating) with a 10 mm diameter artificial vessel (yellow arrow indicating) was made from a bovine pericardial patch (120 × 80 mm). **B** A postoperative computed tomographic scan showing an unobstructed bovine pericardial duct and good reconstruction shape of the left subclavian artery by a 10 mm branch

Surgery was performed in the right decubitus position through the fourth intercostal approach with the left posterolateral incision, under deep hypothermic circulatory arrest with the left femoral artery, and left femoral vein cannulation. After the patient cooled down to 20 °C, a deep hypothermic circulatory arrest occurred, the distal arch and proximal descending aorta were opened and inspected, and the anastomotic margin of the arch just distal to the left common carotid artery was trimmed. During the operation, there were unclean secretions in the pseudoaneurysm, the pseudoaneurysm body showed ulcerative manifestations, and the adventitia adhered to the surrounding tissues. The pseudoaneurysm and its contents were excised till the adjacent normal artery wall, and those closely adhered to the surrounding tissues that cannot be removed shall be cauterized with electrocoagulation and disinfected with iodophor. The proximal end of the self-made bovine pericardial (Guanhao Biotechnology Co., Ltd.) duct with a 10 mm single artificial branch (Fig. 2A) was anastomosed with the distal aortic arch. The 10 mm single branch was connected to the arterial perfusion tube to resume cardiopulmonary bypass and start rewarming. The distal end of the bovine pericardial duct is anastomosed to the distal end of the descending aorta. The 10 mm single-branch artificial vessel was anastomosed to the left subclavian artery. The cardiopulmonary bypass and deep hypothermic circulatory arrest times were 140 and 27 min, respectively. The patient recovered uneventfully. The duration of mechanical ventilation support was 16.72 h. The duration of the intensive care unit stay was 83.87 h. The postoperative hospital stay was 7 days. Detection of PD-seq pathogenic macrogenomic DNA and RNA of fresh-frozen surgical tissues of thoracic aortic pseudoaneurysm indicated *Brucella melitensis* infection.

Postoperative CT showed that the bovine pericardial duct was unobstructed, and the left subclavian artery reconstructed by a single branch was good (Fig. 2B). At post-discharge, doxycycline and rifampicin oral antibiotics were continuously administered. The patient did not have any further symptoms of infection such as fever during the follow-up. The patient had hoarseness before the operation, and there was hoarseness after the operation without aggravation.

## Discussion

Brucellosis is a zoonotic disease involving multiple systems, however, cardiovascular involvement is less common [1]. Aortic complications of brucellosis are rare, and thoracic aortic pseudoaneurysm is extremely rare [2]. According to a meta-analysis study on brucellosis in the Chinese population, cardiovascular diseases were reported as 9% [3]. If a pseudoaneurysm is not treated in time, it may rupture at any time; therefore, it must be operated on immediately.

The patient in this report had brucellosis 8 years ago, recovered after treatment, and relapsed again, resulting in pseudoaneurysm formation in the proximal descending thoracic aorta. Surgery was immediately performed as soon as the existing infection was managed. The distal arch and proximal descending aorta were replaced by a self-made bovine pericardial duct, and the left subclavian artery was replaced with a 10 mm artificial vessel. The bovine pericardial conduit was chosen instead of the artificial blood vessel, and biomaterials may have a better anti-infection ability. Maximilian Kreibich mentioned that orthotopic aortic reconstruction using bovine pericardial tube grafts to treat infectious aortic disease is possible in any aortic segment [4]. *Brucella melitensis* infection was confirmed by gene sequencing of postoperative pathological specimens. Antibiotic therapy was continued for 6 postoperative weeks. The patient achieved good short-term results.

Herein, we reported an extremely rare case of descending aortic pseudoaneurysm caused by *Brucella melitensis* infection. A self-made bovine conduit was used to replace the descending aorta and achieve good short-term results. This treatment method has not been reported in the literature, and its long-term efficacy should be further followed up.

## Data Availability

The datasets used and/or analyzed during the current study are available from the corresponding author upon reasonable request.
